# Sensing core histone phosphorylation — A matter of perfect timing^☆^

**DOI:** 10.1016/j.bbagrm.2014.04.013

**Published:** 2014-08

**Authors:** Anna Sawicka, Christian Seiser

**Affiliations:** Department of Medical Biochemistry, Max F. Perutz Laboratories, Medical University of Vienna, Vienna Biocenter, Vienna, Austria

**Keywords:** Histone phosphorylation, Histone code, Transcription, Mitosis, DNA damage

## Abstract

Systematic analysis of histone modifications has revealed a plethora of posttranslational modifications that mediate changes in chromatin structure and gene expression. Histone phosphorylation is a transient histone modification that becomes induced by extracellular signals, DNA damage or entry into mitosis. Importantly, phosphorylation of histone proteins does lead not only to the binding of specific reader proteins but also to changes in the affinity for readers or writers of other histone modifications. This induces a cross-talk between different chromatin modifications that allows the spatio-temporal control of chromatin-associated events. In this review we will summarize the progress in our current knowledge of factors sensing reversible histone phosphorylation in different biological scenarios. This article is part of a Special Issue entitled: Molecular mechanisms of histone modification function.

## Highlights

• Signal induced histone phosphorylation is associated with local chromatin opening and transcriptional activation.
• Histone phosphorylation is also linked with chromatin condensation during mitosis.
• Histone phosphorylation marks are important for regulation of the DNA damage response.
• Specific reader proteins recognize histone phosphorylation marks alone or in combination with other histone modifications.
• Histone phosphorylation affects the affinity of readers or writers of other histone modifications.

## 1 Introduction

Eukaryotic DNA is organized in a complex with histone proteins as chromatin. The nucleosomal (and higher order) chromatin structure facilitates the packaging, organization and distribution of eukaryotic DNA but has a negative impact on several fundamental biological processes such as transcription, replication and DNA repair by restricting the accessibility for high molecular weight protein complexes. Posttranslational modification (PTM) of histones by acetylation, methylation, ubiquitination or phosphorylation has been shown to modulate the chromatin structure by changing protein–DNA or protein–protein interactions. Mass spectrometry analysis and application of modification-specific antibodies led to the identification of a large number of different PTM sites, located at the N-terminal tails as well as within the globular domains of histone proteins [1], [2], [3], [4]. Some of these modifications such as histone methylation at K9 or K27 are more stable PTMs and are crucial for development, heterochromatic silencing and maintenance of cell identity [5]. Other modifications including histone acetylation and phosphorylation are transient and dynamic events [6], [7] and constitute integral components of the chromatin signaling pathway [5]. PMTs of histones, alone or in combination, reflect specific biological events and chromatin states. Reader proteins with particular binding modules recognize specific histone marks and act together with associated complexes to orchestrate a variety of chromatin-associated processes such as transcriptional regulation, chromatin condensation or DNA damage repair [3].

Histone phosphorylation is targeted to serines (S), threonines (T) and tyrosines (Y) and its abundance can range from targeting a minute fraction of nucleosomes during the G0/G1 of the cell cycle [8] to association with most nucleosomes of the G2/M-phase chromatin [9]. Histone phosphorylation marks play an important role in the interpretation of combinatorial PTMs by components of the chromatin-based signaling machinery. In this review we will discuss the function of sensors of histone phosphorylation in the context of transcriptional regulation by extracellular signals, chromatin condensation during mitosis and DNA damage.

## 2 14–3–3 proteins as readers of the H3S10ph mark

**Fig. 1:**
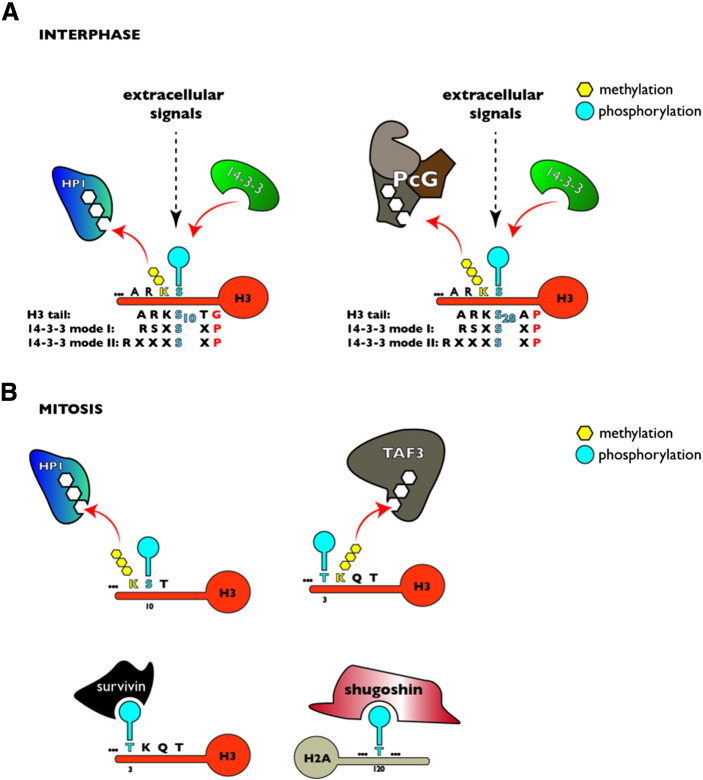
Sensors of histone phosphorylation during interphase and mitosis. (A) The phospho-methyl switch during interphase: Signal-dependent phosphorylation of H3S10 or H3S28 triggers the dissociation of the repressive readers HP1 and PcG from the respective neighboring methylated lysine residues and recruitment of the reader 14–3–3 resulting in the transient activation of target genes. Sequence alignment of high affinity 14–3–3 consensus motifs of mode I and mode II with 14–3–3 binding sites within histone H3. The critical position at P + 2 (red residues) from the phosphorylated serines (blue residues) is occupied by glycine 12 for serine 10 and proline 30 for serine 28, respectively. (B) Redistribution of the chromosomal passenger complex (CPC) and transcriptional silencing during mitosis. Aurora B-mediated phosphorylation of H3S10 during mitosis leads to dissociation of HP1 from H3K9me3. CPC relocalization to the inner centromere is mediated by binding of survivin to H3T3Tph and interaction of borealin-associated shugoshins with phosphorylated H2AT120. H3T3 phosphorylation by haspin results in dissociation of TAF3 from trimethylated H3K4.

Activation of signaling cascades in response to stress, growth factors or immune stimulation ultimately results in the phosphorylation of many cellular targets including histone proteins. Although histone phosphorylation has been studied since the sixties of the last century, only few proteins directly binding this modification have been identified [10], [11], [12], [13]. The discovery of members of the 14–3–3 family as the first selective phospho-histone interacting proteins with specificity for the H3S10ph mark has paved the way to understand the role of this modification in transcriptional activation [14]. 14–3–3 proteins constitute an abundant family of phosphoserine/phosphothreonine binding modules that homo- and heterodimerize to associate with other factors to alter their conformation, cellular localization, enzymatic activity or the ability to interact with other partners [15]. 14–3–3 proteins are highly conserved and are able to complement for the loss of their homologues even when expressed in distantly related species [16]. The mammalian 14–3–3 family comprises seven members that have been demonstrated to interact with 700 different factors [17], including many transcriptional regulators and chromatin-modifying proteins, such as the TATA-binding protein [18], p53 [19] and histone deacetylases [20]. In vitro pull down assays using synthetic peptides corresponding to the N-terminal tail of histone H3 phosphorylated at S10 and human nuclear extracts, followed by mass spectrometry, identified 14–3–3 isoforms as phospho-specific binding proteins [14], [21], [22]. Importantly, the affinity of 14–3–3 for the H3S10ph mark is increased when one of the neighboring lysine residues, K9 or K14 is acetylated [21], [22]. Structural and biochemical studies have revealed the molecular bases of this phenomenon. First of all, the motif containing phosphorylated S10 at histone H3 does not match the known 14–3–3 consensus binding motifs, as it lacks the proline residue at the position P + 2 [15], [23] (Fig. 1A). In agreement with this finding, H3G12P substitution significantly increased the affinity of 14–3–3 to the level observed for a phosphoacetylated H3S10phK14ac peptide in in vitro binding assays [23]. It has been therefore suggested that the presence of H3K14 acetylation counterbalances the lack of proline in the 14–3–3 binding motif. In recent molecular modeling approaches 14–3–3ζ has been shown to preferentially bind the H3S10phK14ac mark and additional acetylation of H3K9 favors binding of the mitogen-activated protein kinase phosphatase-1 (MKP1) to dephosphorylate H3S10 [24]. This in silico analysis demonstrated a preferential interaction of mitogen- and stress-activated kinase-1 (MSK1) with non-acetylated histone H3 compared to K9- and K14-acetylated H3. In summary these data indicate that combinatorial phosphorylation and acetylation of histone H3 modulate the affinity for readers and potentially also for erasers.

## 3 Link between histone phosphorylation and acetylation

Site-directed chromatin immunoprecipitation (ChIP) analysis using dual modification specific antibodies revealed the presence of the H3S10phK14ac mark at activated promoters in vivo [21], [25], [26], [27]. This raises an important question about the mechanism underlying the simultaneous targeting of the two PTMs to the same histone H3 tail. Literature provides two models explaining this phenomenon. The first one implies that histone H3 phosphorylation and acetylation are spatially linked but independent processes and one of the PTMs is not required for the establishment of the other [8], [28]. According to the alternative model, the co-existence of the two PTMs is the result of synergistically coupled recruitment of kinases and histone acetyltransferases. In agreement with this model, the activity of yeast histone acetyltransferases (Gcn5, PCAF and p300) towards K14 on H3 peptides was shown to be significantly higher when a peptide phosphorylated at S10 was used as a substrate [25], [29]. This effect was abolished when R164 of yeast Gcn5, a residue adjacent to H3S10 in Gcn5/CoA/histone H3 complex, was mutated to alanine [29]. This finding, however has been challenged by in vitro assays employing the entire SAGA complex and not only Gcn5, where S10 phosphorylation did not stimulate acetylation of histone H3 peptides [30], [31]. In addition, a recent study employing time-resolved high-resolution NMR spectroscopy also did not report an increased K14 acetylation of H3S10 phosphorylated peptides by recombinant full-length Gcn5 [32]. Importantly however, S10A substitution in histone H3 as well as R164A point mutation in Gcn5 reduced the transcriptional activity of the same set of promoters in yeast, further suggesting that the phosphorylation of S10 is a prerequisite for the acetylation of K14 at histone H3 in vivo [29]. However, this mechanism was shown to operate in a promoter-specific manner [33], [34]. Yeast *GAL1* and *INO1* promoters both require histone phosphorylation and acetylation for TBP recruitment and transcriptional activation, but the establishment of the two marks differs for those promoters. The Gal4 transcription factor recruits both the SAGA complex and the H3S10 kinase Snf1 to the *GAL1* promoter. In the case of the *INO1* promoter, Ino2 transcription factor recruits Snf1 but not the histone acetyltransferase complex. Moreover, Snf1 kinase activity was required for H3K14 acetylation at this promoter [33], [34]. Those findings indicate that both models explaining the coexistence of phosphorylation and acetylation at the histone H3 tail are supported in vivo, depending on the promoter investigated. Importantly, simultaneous phosphorylation of S10 and acetylation of K14 at histone H3, resulting in 14–3–3 binding were shown to be required for full transcriptional activation of *Hdac1* [21] and *p21* [27] genes as well as for VL30 transposable elements [26] upon stress stimulation in the presence of HDAC inhibitors. In addition, 14–3–3 binding to phosphorylated histone H3 was shown to impact on the acetylation status of lysine residues at other histone tails. Upon serum stimulation, the PIM1 kinase phosphorylated K9-acetylated histone H3 tails at the FosL1 enhancer, resulting in binding of 14–3–3. In turn, 14–3–3 recruited the histone acetyltransferase MOF, which modified H4K16, resulting in BRD4 binding and subsequent P-TEFb recruitment. This complex sequence of chromatin modification events finally triggered the release of RNA Polymerase II from the promoter proximal state and transcriptional activation of the FosL1 gene [35]. A similar mechanism was described in *Drosophila*, where 14–3–3 recruited Elongator protein 3 (Elp3), an acetyltransferase active during transcriptional elongation targeting H3K9 [36].

Interestingly, H3S10 and H3S28 reside within the same -ARKS- motif (Fig. 1A). Moreover, in vitro peptide binding assays demonstrated that the affinity of 14–3–3 for S28 phosphorylated peptides and H3S10K14 phosphoacetylated peptides is very similar [23]. This is due to the fact that H3S28 is followed by a proline residue at the position P + 2, matching the consensus binding motif for 14–3–3 (Fig. 1A). In agreement with these in vitro data 14–3–3 was shown to bind with H3S28ph-associated nucleosomes at immediate early genes [37]. Interestingly, the human histone isoform H1.4 is phosphorylated at S27 within the ARKS motif [38]. It is currently unknown whether 14–3–3 also binds phosphorylated histone H1.4.

Of note, histone phosphorylation does not only impact on the acetylation status of neighboring lysine residues, but also demonstrated to influence the phosphorylation of adjacent serines or threonines. Time-resolved high-resolution NMR spectroscopy studies showed that recombinant Chk1 kinase is unable to phosphorylate H3T11 of histone H3 peptide when S10 is already phosphorylated [32]. However, H3T11 phosphorylation does not impair Msk1 and Aurora B activity towards H3S10. The activity of those kinases was also not affected by the presence of H3T6 phosphorylation, whereas S10 phosphorylation abolished H3T6 phosphorylation by PKCα [32]. These findings indicate that the presence of histone phosphorylation dictates the hierarchy of subsequent histone PTMs, possibly providing a mechanism to define the precise spatio-temporal histone phosphorylation patterns observed in many cellular processes.

## 4 Transient loss of memory — the ‘phospho-methyl switch’

The combinatorial nature of a histone code has led to the hypothesis that dynamic histone phosphorylation at S10 and S28 might affect the readout of stable methylation marks at the respective neighboring residues K9 and K27 [39]. Such a ‘phospho-methyl switch’ mechanism could operate to enable rapid changes in binding of effector proteins to histone PTMs whose turnover rate is very slow. Indeed, two independent studies have demonstrated that the mitotic release of heterochromatin protein 1 (HP1) proteins α, β and γ from pericentric chromatin results from Aurora B-mediated phosphorylation of histone H3 at S10 [9], [40] (Fig. 1B). HP1 proteins play an important role in heterochromatin organization and their association with chromatin is primarily mediated by the interaction of their chromodomains with trimethylated H3K9 [41], [42]. In the absence of mitotic phosphorylation, either due to the chemical inhibition of Aurora B with hesperadin or siRNA-mediated knockdown of Aurora B, HP1 α, β and γ remained localized at the condensed chromatin [9], [40]. Moreover, fluorescence polarization measurements demonstrated a significant reduction in affinity of both HP1 chromodomains and full-length recombinant proteins to the H3K9me3 peptide, when adjacent S10 was phosphorylated [9]. The interaction between HP1 and methylated H3K9 is mediated by the conserved glutamic acid residue located within a chromodomain groove of HP1, as substitutions at this site affected HP1 binding to H3K9 methylated peptides [9], [43]. In addition, H3S10 phosphorylation is able to disrupt HP1 interaction with methylated H3K9 peptide in in vitro kinase assays [9]. Although recombinant Ipl1/Aurora kinase exhibits significantly reduced activity towards an H3K9me2 peptide in comparison to an unmodified one [44], the chromosomal passenger complex (CPC) [45], containing Aurora B with its activating factors, efficiently phosphorylates H3S10 in the presence of trimethylated K9, leading to dissociation of HP1 [9]. Yet, mitotic release of HP1 from chromatin in vivo might require additional histone PTMs and/or chromatin-associated factors. For instance, Mateescu et al. reported that acetylation of K14 at histone H3, in addition to H3S10ph at the same histone H3 tail, is needed to dissociate HP1α from chromatin during G2/M transition [46]. Using overlay assays in the presence of differentially modified histone H3 peptides the authors found that the presence of S10 phosphorylation stabilizes the binding of recombinant HP1α to histone H3 tail, whereas H3K14 acetylation, which occurs after H3S10 phosphorylation, abolishes HP1–H3 interaction. Interestingly, another study, applying a quantitative proteomics approach, reported only minor influence of H3S10ph on HP1 binding to H3K9me3 [47]. However the authors did not address possible consequences of K14 acetylation for this interaction. The discrepancies between the studies indicate that biological consequences of H3S10ph in the context of HP1 binding to H3K9me3 in vivo are highly dependent on the cellular context and are regulated by a multi-layered cross-talk between histones, histone modifying enzymes and other chromatin-associated factors. Importantly, 14–3–3 proteins do not associate with mitotic chromosomes [14], [21], signifying a cell cycle-dependent interpretation of histone phosphorylation marks. Regardless of the requirement of additional factors, H3S10ph-induced HP1 dissociation during mitosis does not alter H3K9me3 levels [9], constituting an efficient mechanism to maintain the epigenetic memory throughout cell division that allows to rapidly re-establish the repressive state in daughter cells. Strikingly, this seems to be a more frequent phenomenon since another mechanism employing the ‘phospho-methyl switch’ during mitosis has been reported. Similarly to H3K9me3, H3K4me3 levels are stably maintained during mitosis, which was suggested to ‘bookmark’ genes for immediate activation once mitosis has been completed [48]. H3K4me3 is primarily found at transcriptionally active promoters and is specifically recognized by a plant homeodomain (PHD) finger of the TAF3 subunit of the TFIID complex, facilitating the recruitment of the basal transcriptional machinery to active chromatin [49], [50]. Upon entry into mitosis, actively transcribed genes become silenced, coinciding with the dissociation of TFIID from mitotic chromatin [51], [52] (Fig. 1B). Interestingly, phosphorylation of H3T3 interferes with binding of TAF3-PHD to an H3K4me3 peptide in vitro [53], consistent with the NMR structure of TAF3-PHD/H3K4me3, where H3T3 is localized inside the PHD domain [54]. Furthermore, overexpression of haspin, a mitotic H3T3 kinase, decreased the level of chromatin-bound TFIID, whereas haspin knockdown resulted in increased association of TFIID with mitotic chromosomes [53]. These data demonstrate a crucial role of haspin-mediated H3T3 phosphorylation in the timing of TFIID interaction with chromatin during the cell cycle.

## 5 Cross-talk during transcriptional regulation

Of note, the ‘phospho-methyl switch’ is not only restricted to mitosis, but also operates in response to extracellular signaling. For instance, in response to stress activation of the MAPK pathway, H3S10 becomes phosphorylated by MSK1/2 kinases, triggering the dissociation of HP1γ from H3K9me2-modified nucleosomes at the *Hdac1* promoter [21]. Similar to the situation during mitosis, HP1 release does not affect the level of H3K9me2, constituting a convenient mechanism to transiently relieve the repression of specific target genes (Fig. 1A). Since MAPK-stimulated H3S10 phosphorylation has been implicated in the transcriptional induction of many genes, future ChIP-seq studies employing dual specificity antibodies recognizing the H3K9me2S10ph mark should clarify whether the ‘phospho-methyl switch’ is a general mechanism to transiently overcome HP1-mediated gene repression.

Importantly, the association of HP1α with nucleosomes is mediated not only by its chromodomain recognizing methylated H3K9, but also by the carboxy-terminal chromo-shadow domain [55]. HP1α interacts in vitro with an H3 peptide encompassing residues 31–56 and phosphorylation of the Y41 residue abolishes this binding [56]. H3Y41 is a target of the non-receptor kinase JAK2, which is often mutated in hematological disorders. JAK2-mediated H3Y41 phosphorylation resulted in the displacement of HP1α from the *lmo2* promoter and subsequent activation of this oncogene [56]. Genome-wide analysis of H3Y41 phosphorylation in human erythroid leukemia cells by ChIP-seq revealed the enrichment of this mark at a subset of active promoters, at distal cis-regulatory elements overlapping with STAT5 binding and in transcribed regions of tissue-specific genes [57]. Both receptor- and non-receptor tyrosine kinases are found in the nucleus and their abundance is often increased in various malignancies [58]. Therefore, a changed gene expression program linked to dissociation of HP1α upon H3Y41 phosphorylation might be a more frequent mechanism in cancer.

Interestingly, tyrosine phosphorylation at histones has been also demonstrated to attenuate transcription. During the late S phase of the cell cycle the nuclear kinase WEE1 phosphorylates histone H2B at Y37 upstream of the *Hist1* histone gene cluster [59]. H2BY37 phosphorylation induces the dissociation of Nuclear Protein Ataxia-Telangiectasia Locus (NPAT), an essential activator of histone gene transcription, and RNA polymerase II ensuring the cell cycle phase-specific regulation of histone gene expression.

Both S10 and S28 at histone H3 are targeted by MSK1/2 kinases but it was shown that the two modifications target distinct pools of nucleosomes in the nucleus [60], [61]. However, both modifications are associated with the promoters of the same IE genes upon MAP kinase activation [37]. H3S10ph and H3S28ph reside within the identical -ARKS- motif, indicating that phosphorylation of both sites might have similar mechanistic consequences (Fig. 1A). This aspect is particularly interesting in the context of gene silencing by the polycomb group (PcG) system, as H3S28 is adjacent to H3K27, which in the trimethylated state, is bound by polycomb repressive complexes PRC1 and PRC2. Therefore, a ‘phospho-methyl switch’ could constitute a potential mechanism that regulates the association of polycomb group proteins (PcG) with chromatin. Although the PcG system is required for normal differentiation and maintaining cellular identity [62], [63], it is still poorly understood how extracellular signaling regulates PcG target genes. Interestingly, in vitro peptide pull down assays using nuclear HeLa extracts demonstrated that PRC2 binding to the H3K27me3 peptide is completely abolished when the neighboring S28 residue is phosphorylated, suggesting that the phosphorylation status of H3S28 can affect PcG binding in vivo [64] (Fig. 1A). Interestingly, MSK1/2-mediated H3S28 phosphorylation was shown to dissociate PcG proteins in response to stress, mitogenic and differentiation signals [64]. The authors generated H3K27me3S28ph-specific antibodies and demonstrated the presence of this mark at PcG-derepressed genes during gene activation by the abovementioned stimuli. These effects were MSK1/2-dependent as chemical inhibition as well as shRNA-mediated knockdown of the kinases greatly reduced the level of H3K27me3S28ph modification, PcG dissociation and transcriptional induction of target genes. Strikingly, upon mitogenic stimulation transient displacement of PcG from the immediate early gene *ATF3* promoter correlated with the presence of the H3K27me3S28ph double mark and transiently induced expression of the gene (Fig. 1A).

## 6 The signal-induced ‘phospho-methyl-acetyl switch’

Recently it was demonstrated that artificial targeting of the MSK1 kinase to the α-globin promoter resulted in transcriptional activation of this PcG target gene [65]. The authors showed that MSK1 fused to the DNA binding domain of NF1 transcription factor binds the NF1 element within the promoter of the α-globin gene and reactivates its expression in non-erythroid cells, where it is normally silenced by PcG [66]. The recruitment of an active, but not a kinase-dead version of the enzyme resulted in phosphorylation of H3S28 at this promoter, PcG dissociation and reduced H3K27me3 levels concomitant with an increase in H3K27 acetylation. Using an H3K27acS28ph-specific antibody the authors demonstrated that H3S28ph induces a “phospho-methyl-acetyl” switch, which can functionally counteract PcG-mediated silencing [65]. Of note, it has been previously reported that loss of PRC2 activity causes a global increase in H3K27 acetylation [67] indicating potential further implications of the ‘phospho-methyl-acetyl switch’ mechanism. The H3S28ph-mediated dissociation of PcG as well as functional coupling of H3K27 acetylation to H3S28 phosphorylation raises the intriguing question whether H3S28ph regulates the demethylation of H3K27. This is particularly interesting in the light of previous studies that have demonstrated the impact of signal-induced phosphorylation of various sites at the histone H3 tail on the methylation status of the neighboring lysine residues. For instance, in the case of androgen signaling, two such phenomena have been observed: PKCβ-mediated H3T6 phosphorylation inhibited demethylation of H3K4me by LSD1 [68], whereas H3T11 phosphorylation by protein-kinase-C-related-kinase (PRK1) facilitated the demethylation of H3K9me by Jumonji-containing-protein 2C (JMJD2C) [69]. In addition, the demethylase activity of JMJD2A towards H3K9me3 is inhibited upon H3S10 phosphorylation [70].

In conclusion, the existing literature clearly demonstrates an important role of histone phosphorylation in the interpretation of other pre-existing histone PMTs located at the same histone tail. For instance, phosphorylation of H3S10 and H3S28 is able to transiently override the repressive effects of the respective neighboring H3K9me2 and HK27me3 marks. Finally, a variation of the ‘phospho-methyl switch’ was recently demonstrated for the epigenetic regulator UHRF1 which links DNA methylation to histone modifications. UHRF1 simultaneously binds by its tandem tudor domain to unmodified H3R2 and via the PHD finger to trimethylated H3K9 [71]. Importantly phosphorylation of H3T3 destabilizes the interaction between the N-terminal tail of H3 with UHRF1, while H3S10 phosphorylation strongly reduces the affinity of TTD-PHD for the H3K9me3 tail. Since many methylated lysine residues of histone H3 are adjacent to phosphorylatable residues, the ‘phospho-methyl switch’ might be a prevalent mechanism in controlling the association of proteins with chromatin in various biological processes.

## 7 Centromere organization and chromatin condensation

The unique spatio-temporal patterns of histone phosphorylation at various residues during mitosis indicate their functions in specific phases of this process and reflect the precise control of the localization and activity of mitotic kinases. Particularly, recent studies shed light on the crucial role of mitotic histone phosphorylation in the regulation of localization and functions of the chromosomal passenger complex (CPC), a key regulator of mitotic events. CPC consists of Aurora B and three regulatory subunits: inner centromere protein (INCENP), survivin and borealin [72]. During interphase HP1 targets CPC to heterochromatin and Aurora B-mediated phosphorylation of H3S10 at the beginning of prophase leads to dissociation of HP1 from trimethylated H3K9 (Fig. 1A), releasing CPC from chromosome arms and thereby enabling its recruitment to the inner centromere [73], [74]. The enrichment of CPC at the inner centromere requires two other mitosis-specific phosphorylation events: H3T3 phosphorylation by Haspin and H2AT120 phosphorylation by Bub1 kinase [45]. Although the individual distributions of these PTMs differ, the inner centromere is defined by the intersection of the two marks, where CPC reaches its maximal concentration [45] (Fig. 1B). CPC associates with H3T3 phosphorylated nucleosomes via the BIR domain of survivin, which binds the three N-terminal amino acids of histone H3 when H3T3 is phosphorylated [75], [76]. Of note, the BIR domain had not been considered as phospho-specific recognition module, as it was described to interact with the N-terminal alanine residue of Inhibitor of Apoptosis (IAP) binding motif (IBM) present in Smac/DIABLO [77]. Phosphorylated H2AT120 recruits shugoshins Sgo1 and Sgo2 that in turn interact with borealin [45], [78] (Fig. 1B). In addition, centromeric regions are marked by H3T11 phosphorylation during metaphase, which was linked to kinetochore assembly [79]. Recent work has defined a long-disputed role of H3S10 phosphorylation in chromatin condensation during mitosis. Using *Saccharomyces cerevisiae* as a model, the authors showed that H3S10 phosphorylation is required for recruitment of the histone deacetylase HST2, which mediates deacetylation of H4K16 [80]. This event, in turn, is required for the interaction of the histone H4 tail with the acidic patch of H2A and subsequent chromatin condensation. Taken together, a cascade of histone phosphorylation events during early mitosis establishes the condensed structure of metaphase chromosomes (Fig. 1B).

## 8 DNA damage response

The cellular metabolism as well as environmental genotoxic agents constantly challenge DNA integrity [81]. DNA double-strand breaks (DSBs) are probably the most dangerous DNA lesions, which, if inefficiently or inaccurately repaired, may cause genetic rearrangements, resulting in cell death or oncogenic transformation [82]. Histone phosphorylation plays an important role in the regulation of DSB response. DSB signaling is triggered by the recognition of free DNA ends by the MRE–RAD50–NBS1 (MRN) complex, which recruits S/T protein kinase (ataxia-telangiectasia mutated), a member of the PI3K-like kinase family [83]. One of the earliest ATM targets is histone H2A.X, whose phosphorylated form at S139 (in higher eukaryotes) or S129 (in yeast), termed γH2A.X, is considered as a hallmark of DSB recognition [84], [85], [86]. The H2A.X histone variant accounts for up to 10% of total histone H2A [87] and the presence of the C-terminal SQEY motif, containing S139, distinguishes it from its canonical counterpart. γH2A.X formation is rapidly induced upon DSB, as it was detected already 3 min after the exposure of breast cancer cells to 0.6 Gy of γ-radiation [88]. Strikingly, H2A.X phosphorylation spreads over 2 Mb regions around DSBs in mammalian cells and up to 50 kb in yeast [86], [89]. ATM is the primary, but not the only kinase targeting H2A.X, as ATR (ATM- and Rad3-related) and DNA-PK (DNA-dependent protein kinase) were also described to phosphorylate this histone variant [90].

**Fig. 2:**
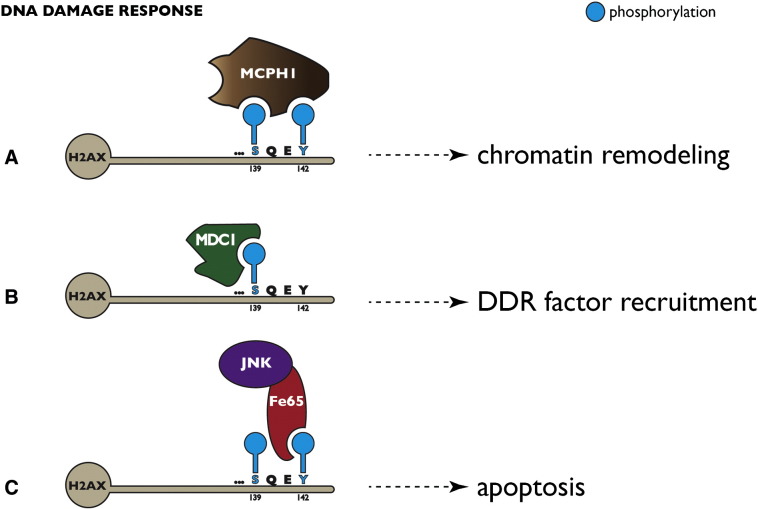
Histone phosphorylation signals during DNA damage response. (A) In an early phase of DNA Damage Response MCPH1 binds to H2AX phosphorylated on S139 and Y142 and recruits the SWI/SNF chromatin remodeling complex. (B) Upon removal of the Y142ph mark by Eya1/Eya3 MDC1 can bind as component of DNA repair complexes to phosphorylated S139 of histone H2AX. (C) Alternatively, the Y142ph mark is maintained and bound by the adapter protein Fe65, which recruits the proapoptotic kinase JNK1.

The importance of H2A.X phosphorylation for DNA Damage Response (DDR) has been demonstrated in loss-of-function studies. Mice deficient for H2A.X are viable but display several abnormalities including accumulation of chromosomal aberrations during M phase, G2/M checkpoint defects and enhanced radio-sensitivity [91], [92], [93]. Importantly, the loss of a single H2A.X allele promotes genomic instability and renders the animals more vulnerable to cancer in the absence of p53 [94]. In addition, restoration of a null allele with an H2A.X version carrying a substitution of conserved S139 with alanine or glutamic acid did not rescue the phenotype, emphasizing the importance of reversible phosphorylation of this site for maintaining genomic integrity [94]. This possibly has important implications for human diseases, since the H2A.X locus localizes to chromosome 11q23.3, a region that is often found deleted in lymphoid and solid tumors [95]. Of note, H2A.X was shown to be dispensable for the initial steps of DDR as the recruitment of downstream factors in DSB signaling cascade was remained unaffected in cells lacking H2A.X as well as in H2A.X-deficient cells reconstituted with H2A.X version carrying S139A or S139E substitutions. However, the retention of those factors at the site of DNA damage depended on the presence of γH2A.X [96]. In vitro pull down assays using nuclear extracts and peptides corresponding to the last 20 C-terminal residues of H2A.X identified Mediator of DNA Damage Checkpoint Protein 1 (MDC1) as a factor binding a phosphorylated (S139ph) but not unphosphorylated version of the peptide [97] (Fig. 2B). Further experiments revealed the tandem BRCT (breast cancer-associated protein carboxy-terminal) domains [98] as binding modules mediating the interaction between γH2A.X and MDC1. The BRCT domain was originally identified in the tumor suppressor protein BRCA1 and is an integral signaling module in many other proteins including factors involved in DDR such as TOPBP1, MCPH1, Rad9, 53BP1, Crb2, NBS1 and Poly(ADP-ribose) polymerase 1 (reviewed in [99], [100]). In addition to their well-known role as phospho-binding modules, BRCT domains have been implicated in phosphorylation independent protein interactions, DNA binding and poly(ADP-ribose) binding. The crystal structure of the BRCT (MDC1)–γH2A.X complex identified three residues (T1898, K1936 and R1933) in the sequence of BRCT crucial for its binding to γH2A.X. The importance of this interaction was confirmed in vivo, as full-length MDC1 protein fused to GFP was able to recruit 53BP1, NBS1 and phosphorylated ATM to the sites of DNA damage, whereas R1933Q and K1936M MDC1 mutants failed to do so [97]. Yet, MDC1 binding to γH2A.X is regulated by another phosphorylation event at Y142, preventing MDC1 from binding to the phosphorylated S139 [101]. Moreover, the phosphorylation state of Y142 plays a very important role in directing the DSB response either towards apoptosis or repair. The multidomain adapter protein Fe65 was shown to bind phosphorylated Y142 at H2A.X via its PTB2 domain (Fig. 2C). Fe65 serves as an adapter protein recruiting proapoptotic kinase JNK1. Importantly, mouse embryonic fibroblasts expressing a Y142F mutant of H2A.X displayed a reduced apoptotic response to high-dose ionizing radiation in comparison to wild-type cells [102], suggesting that the absence of phosphorylated Y142 promotes DNA damage repair due to MDC1 recruitment instead of a proapoptotic response upon genotoxic stress. Interestingly, at early stages of DDR, phosphorylated Y142 recruits MCPH1 (microcephalin), which in turn interacts with the SWI/SNF chromatin remodeling complex via ATM/ATR-mediated phosphorylation of the BAF70 subunit [103] (Fig. 2A). In agreement with this finding, MCPH1-deficient cells fail to repair DNA lesions as a result of decreased association of SWI/SNF complex and impaired chromatin relaxation at the sites of DNA damage [103]. The role of γH2A.X in the context of Y142 phosphorylation provides another interesting example how spatio-temporal changes in histone phosphorylation patterns regulate chromatin-templated processes. Under basal conditions Y142 is constitutively phosphorylated by the WSTF (Williams syndrome transcription factor) kinase. In response to DSBs, H2A.X becomes phosphorylated at S139 and progressively dephosphorylated at Y142 by Eya1 and Eya3 tyrosine phosphatases enabling MDC1 binding [102]. Elegant biochemical and structural studies have provided insight into mechanistic aspects of the recognition of those differential phosphorylation patterns by effector proteins. Neither MDC1 nor MCPH1 is able to bind H2A.X-derived peptides carrying the Y142ph mark alone. Interestingly, MCPH1 is able to bind doubly phosphorylated peptides (phosphorylated at Y142 and S139) with its tandem BRCT domains, however its affinity is substantially higher when the substrate is phosphorylated only at S139, whereas MDC1 is unable to bind doubly phosphorylated peptides [104]. In a recent report [105], another DNA damage-dependent histone phosphorylation mark was characterized in yeast. The checkpoint protein kinases Tel1 (ATM in mammals) and Mec1 (ATR in mammals) phosphorylate histone H2B at T129 forming γ-H2B in response to DSBs. γ-H2B formation is impaired by γ-H2AX and its binding partner Rad9 which binds to γ-H2AX through its BRCT domains. Taken together these findings explain how the kinetics of histone phosphorylation dictates the order of events at the sites of DNA damage.

Recent work has revealed a role of H3S10 phosphorylation in transcription-associated genomic instability as a result of R loop accumulation [106]. R-loops are triple-stranded nucleic acid structures formed co-transcriptionally by an RNA:DNA hybrid and a displaced non-template DNA strand. R-loops occur naturally during replication of bacterial plasmids and mitochondrial DNA as well as during class-switching recombination in B cells. They are also formed as transcription byproduct and, if not efficiently removed, can threaten genome integrity [107]. In particular, R-loops may induce replication fork stalling, transcription–replication collisions and DSB formation. Only recently it was observed that in *S. cerevisiae* the R-loop accumulating *hpr1Δ* strain shows increased levels of H3S10ph, particularly at centromeric and pericentromeric regions and to a smaller extent across open-reading frames. The R-loop-induced H3S10 phosphorylation linked to chromosome condensation was also observed in *Caenorhabditis elegans* and human cells suggesting a conserved nature of this mechanism. The authors propose the model in which R-loop formation induces H3S10 phosphorylation and chromatin compaction, which in turn may result in transcription–replication collisions and promote genomic instability [106].

## 9 Conclusions

A unique set of features characterizing histone phosphorylation distinguishes it from other chromatin modification pathways. The abundance of specific histone phosphorylation marks can vary dramatically between interphase and mitosis [9] and it is likely that individual histone phosphorylation marks have different half-lives. It will be important to address these issues using quantitative proteomics approaches. To date only few reader proteins, mainly members of the 14–3–3 family and BRCT proteins have been identified. In contrast to readers of other histone PTMs 14–3–3 proteins are not typical chromatin-associated factors but rather components of the signaling machinery recognizing a large number of S/T-phosphorylated proteins.

The interpretation of this modification is highly context-dependent, as phosphorylation of the particular residue at different stages of the cell cycle results in completely different and often opposite biological effects. In addition, the transient nature of histone phosphorylation allows for temporal changes in the interpretation of neighboring stable histone modification. This is particularly important in the context of extracellular signaling, as it enables a rapid transcriptional response while maintaining cellular identity. Employing dual modification antibodies for phosphoacetylated or phosphomethylated histones in genome-wide ChIP-seq analysis should reveal the importance of these combined histone marks for gene regulation. Finally, recent advances in mass spectrometry approaches [108] will definitely contribute to the identification of additional reader proteins also for combinatorial modifications and might lead to the discovery of novel functions of histone phosphorylation marks in chromatin templated processes.
